# Isolation and identification of a phytotoxic substance from the emergent macrophyte *Centrostachys aquatica*

**DOI:** 10.1186/s40529-014-0059-1

**Published:** 2014-08-12

**Authors:** Tran Thi Ngoc Bich, Hisashi Kato-Noguchi

**Affiliations:** 1 Department of Applied Biological Science, Faculty of Agriculture, Kagawa University, Miki, 761-0795 Kagawa Japan; 2 Department of Agriculture, The South College of Engineering and Agriculture, Cần Thơ, Vietnam

**Keywords:** Allelopathy, Bioactive substance, Centrostachys aquatica, Loliolide, Macrophyte, Phytotoxicity

## Abstract

**Background:**

*Centrostachys aquatica* is a perennial emergent macrophyte in marshy places and in rivers. The species was recorded in Senegal and Nigeria, but widespread in tropical Africa, and South and East Asia. Aqueous methanol extracts *C. aquatica* was found to be toxic to several plant species. However, no phytotoxic substance has been reported in this species. Therefore, we investigated phytotoxic activity and searched for phytotoxic substances with allelopathic activity in *C. aquatica.*

**Results:**

An aqueous methanol extract of *C. aquatica* inhibited the growth of roots and hypocotyls of cress (*Lepidium sativum*). The extract was then purified by several chromatographic runs and a phytotoxic substance with allelopathic activity was isolated and identified by spectral analysis as loliolide. Loliolide inhibited cress root and hypocotyl growth at concentrations greater than 0.03 μM. The concentrations required for 50% growth inhibition of cress roots and hypocotyls was 0.18 and 0.15 μM, respectively.

**Conclusion:**

These results suggest that loliolide is a phytotoxic substance and may contribute to the allelopathic effect caused by *C. aquatica.*

**Electronic supplementary material:**

The online version of this article (doi:10.1186/s40529-014-0059-1) contains supplementary material, which is available to authorized users.

## Background

*Centrostachys aquatica* (R.Br.). Wall ex Moq Tand (Synonym, *Achyranthes aquatica* R.Br; Amaranthaceae) is a perennial emergent macrophyte with 0.3-1.5 m stem in length and rooting at nodes forming meadows in marshy places and in rivers. The species was recorded from Senegal and Nigeria, but widespread in tropical Africa, and South and East Asia including India (Baker [1913]; Townsend [1988]).

Many exotic plants naturalized in invasive areas due to their special life-history traits, such as high rate of growth and reproduction, and phenotypic plasticity (Mack [1996]; Cappuccino and Arnason [2006]). High defense capacity of the invasive plants against pathogens and herbivores is also very critical in the new habitats (Keane and Crawley [2002]; Mitchell and Power [2003]; Cappuccino and Carpenter [2005]). In addition, some invasive plants contain large amounts of phytotoxic and/or allelopathic substances, which are toxic to native plant species in the invasive areas (Callaway and Ridenour [2004]; Chengxu et al. [2011]). *Centaures maculosa* releases an allelopathic substance, catechin, which makes its invasion into the new habitats (Callaway and Aschehoug [2000]). Thus, the production of allelopathic and phytotoxic substances may be one of the important traits for invasive plants for the new habitats (Meiners et al. [2012]).

It was also reported that aqueous methanol extracts of *C. aquatica* were toxic to several plant species and shown allelopathic activity (Bich and Kato-Noguchi [2012]). The findings suggest that *C. aquatica* may contain phytotoxic substances with allelopathic activity. However, no such substance has been reported in *C. aquatica* so far. The objective of this study was the isolation and identification of phytotoxic substances with allelopathic activity in this species.

## Methods

### Plant materials

Whole plants of *Censtrotachys aquatica* (R.Br.) Wall ex Moq Tand were collected from riversides, in Ô môn district, Cần Thơ City, South of Vietnam (9°27′ N, 106° E), in January of 2012 (dry season), washed with tap water and dried under sunlight. Dry materials were then packed and protected from air humidity by a silica gel desiccant and stored at 3°C until extraction. Cress (*Lepidum sativum* L.), obtained from Takii Co. (Kyoto, Japan), was chosen as a test plant for the bioassays due to their known seedling growth behavior.

### Extraction

Dried plants of *C. aquatica* (300 g) were soaked in 1.5 L of 70% (v/v) aqueous methanol for two days. After filtration using filter paper (No. 2; Toyo, Tokyo, Japan), the residue was soaked again in 500 mL methanol for two days and filtered, and the two filtrates were combined. An aliquot of the extract (final assay concentration of tested samples corresponded to the extracts obtained from 0.01, 0.03, 0.1 and 3 g dry weight of *C. aquatica* per mL) was evaporated to dryness and biological activity of the extracts was determined by a cress bioassay as described latter.

### Separation of the aqueous methanol extract

Dried plants of *C. aquatica* were extracted as described above. The two filtrates were combined and concentrated at 40°C in vacuo to produce an aqueous residue. The aqueous residue was then adjusted to pH 7.0 with 1 M phosphate buffer, partitioned three times against an equal volume of ethyl acetate, and separated ethyl acetate and aqueous phase. The biological activity of the aqueous and ethyl acetate fractions was determined using a cress bioassay as described latter.

### Isolation of active substance in ethyl acetate fraction

The ethyl acetate fraction was evaporated to dryness and separated on a column of silica gel (80 g, silica gel 60, 70-230 mesh; Merck), eluted with 20, 30, 40, 50, 60, 70 and 80% ethyl acetate in *n*-hexane (100 mL per step), ethyl acetate (100 mL) and methanol (200 mL). The biological activity of all separated fractions was determined using a cress bioassay as described latter, and the most active fraction was obtained by elution with 70% ethyl acetate in *n*-hexane.

After evaporation of the most active fraction, the residue was purified by a column of Sephadex LH-20 (60 g, Amersham Pharmacia Biotech, Buckinghamshire, UK), and eluted with 10, 20, 30, 40, 60 and 80% (v/v) aqueous methanol (100 mL per step), and methanol (200 mL). The active fraction was eluted by 30% aqueous methanol. After evaporation, the residue was dissolved in 20% (v/v) aqueous methanol (2 mL) and loaded onto reverse-phase C_18_ cartridges (YMC Ltd., Kyoto, Japan). The cartridge was eluted with 20, 40, 60, 80 and 90% (v/v) aqueous methanol, and methanol (15 mL per step). The active fraction was eluted by 80% aqueous methanol and evaporated to dryness. The residue was finally purified by reverse-phase HPLC (10 mm ID × 500 mm; YMC- Pack ODS AQ-325; YMC Ltd.) eluted at flow rate of 1.5 mL min^−1^ with 40% aqueous methanol and detected at 220 nm. The inhibitory activity was detected at a peak of 162 -172 min, yielding active compound as colorless oil. The compound was characterized by HRESIMS, ^1^H-NMR spectrum (400 MHz, CD_3_OD) and the specific rotation.

### Cress bioassay for *C. aquatica* extract and HPLC-purified compound

Test samples (aqueous methanol extracts and a purified compound) were dissolved in a 0.2 mL of methanol and added to a sheet of filter paper (No. 2) in a 3-cm Petri dish. The methanol was evaporated in a fume hood. Then, the filter paper in the Petri dishes was moistened with 0.8 mL of 0.05% (v/v) polyoxyethylene sorbitan monolaurate (Tween 20). Ten seeds of cress were placed in the Petri dishes. The length of roots and hypocotyls of the seedlings were measured after 48 h of incubation in the darkness at 25°C, and compared to control seedlings. Controls were treated exactly as described above, with the exception that 0.2 mL methanol was used instead of *C. aquatica* extract or isolated compound. The bioassay was repeated three times using a randomized design with 10 plants for each determination. Significant differences between control and treatment were examined by Welch’s *t*-test. Significant differences among treatments were examined by Duncan’s multiple comparison tests.

## Results and discussion

### Inhibitory activity of the extract of *C. aquatica* and separation

**Figure 1:**
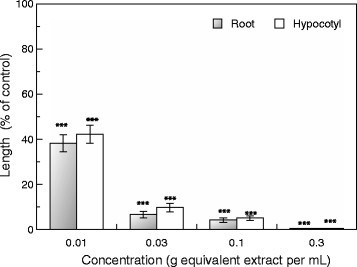
**Effects of aqueous methanol extracts of** ***C. aquatica*** **on root and hypocotyl growth of cress.** Asterisks indicate significant difference between control and treatment: ***, P < 0.001.

The aqueous methanol extract of *C. aquatica* inhibited root and hypocotyl growth of cress seedlings. Increasing the extract concentration resulted in an increase in the inhibition (Figure 1). The extract obtained from 0.1 g of *C. aquatica* inhibited cress root and hypocotyl growth to 4.2 and 5.1% that of the control, respectively. It has been reported that aqueous methanol extracts of *C. aquatica* had the phytotoxic effect on several plant species including weed plants (Bich and Kato-Noguchi [2012]). These results suggest that the extract of *C. aquatica* may have phytotoxic substances with allelopathic activity.

**Figure 2:**
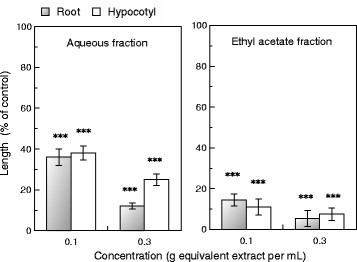
**Effects of aqueous and ethyl acetate fractions separated from the** ***C. aquatica*** **extract on root and hypocotyl growth of cress.** Asterisks indicate significant difference between control and treatment: ***, P < 0.001.

The extract of *C. aquatica* was then divided into aqueous and ethyl acetate fractions. Both fractions inhibited root and hypocotyl growth of cress seedlings (Figure 2). However, inhibitory activity of the ethyl acetate fraction was greater than that of the aqueous fraction. Thus, isolation of phytotoxic substances proceeded using the ethyl acetate fraction.

Then, the ethyl acetate fraction of *C. aquatica* was separated on a silica gel column and the biological activity of all fractions was determined by the cress bioassay. The inhibitory activity was found in the fractions 5, 6 and 7, obtained by elution with 60, 70 and 80% ethyl acetate in *n*-hexane, respectively. The most active fraction 6 inhibited the growth of cress roots and hypocotyls by 7.2 and 13.8% that of control roots and hypocotyls, respectively.

### Identification of a phytotoxic substance

**Figure 3:**
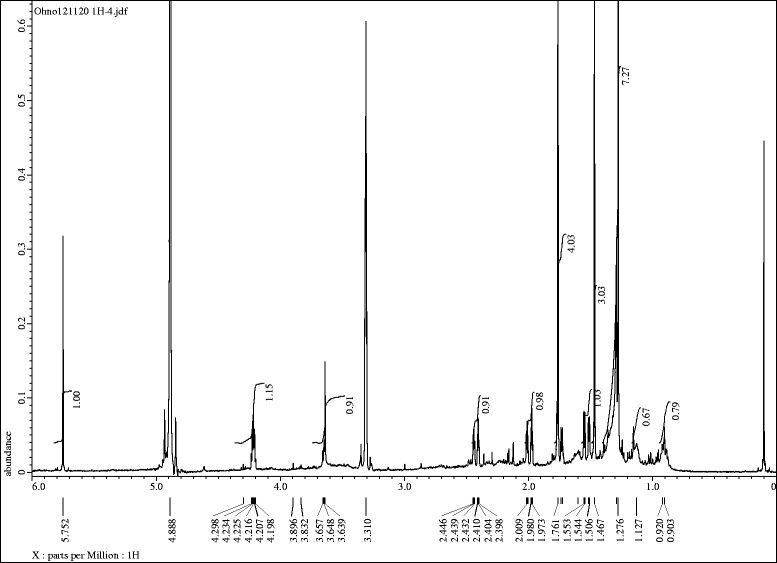
^**1**^ **H NMR spectrum (400 MHz, CD**_**3**_ **OD, TMS as internal standard) of isolated compound.**

**Figure 4:**
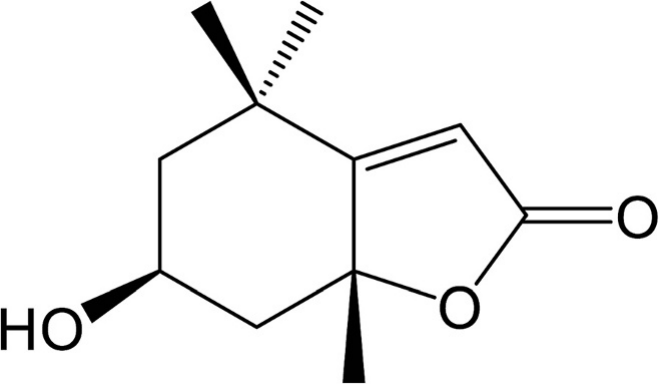
**Chemical structure of loliolide.**

The active fraction 6, eluted with 70% ethyl acetate in *n*-hexane on the silica gel column, was further purified by Sephadex LH-20, reverse-phase C_18_ and HPLC. The biological activity of all fractions after every separation step was determined by the cress bioassay. The most active fraction in each separation step was further purified and an active compound was isolated. The molecular formula of the active compound was determined to C_11_H_20_O_3_ by as suggested by HRESIMS at *m/z* 197.1158 [M + H]^+^ (calcd for C_11_H_17_O_3_, 231.1361, ∆ = -2.0 mmu). The ^1^H spectrum (400 MHz, CD_3_OD, TMS as internal standard, Figure 3) of the compound showed δ_H_ 5.70 (s, 1 H, H7), 4.34 (m, 1 H, H3), 2.46 (ddd, *J* = 14.1, 2.9, 2.4 Hz, 1 H, H4b), 1.98 (ddd, *J* = 14.6, 2.9, 2.4 Hz, 1 H, H2b), 1.79 (dd, *J* = 13.7, 3.4 Hz, 1 H, H4a), 1.79 (s, 3 H, H11), 1.54 (dd, *J* = 14.1, 3.4, Hz, 1 H, H2a), 1.47 (s, 3 H, H9), 1.28 (s, 3 H, H10). The specific rotation of the compound was [α]_D_^22^ = -41° (*c* = 0.06, CHCl_3_). The compound was identified as 3-dihydroxy-3,5,5-trimethylcyclohexylidene-4-acetic acid lactone (loliolide, Figure 4) by comparing those data with those in the literature (Hodges and Porte [1964]; Valdés [1986]; Kimura and Maki [2002]).

Loliolide has been isolated from algal species such as *Gracilaria lemaneiformis* (Lu et al. [2011]), *Undaria pinnatifida* (Kimura and Maki [2002]) and *Sargassum ringgoldianum* (Yang et al. [2011]). The compound has also isolated from terrestrial plant species, *Salvia divinorum* (Valdés [1986]) and *Eucommia ulmoides* (Okada et al. [1994]). However, this is the first report of the presence of loliolide in *C. aquatica* as a phytotoxic substance.

### Phytotoxic activity of loliolide

**Figure 5:**
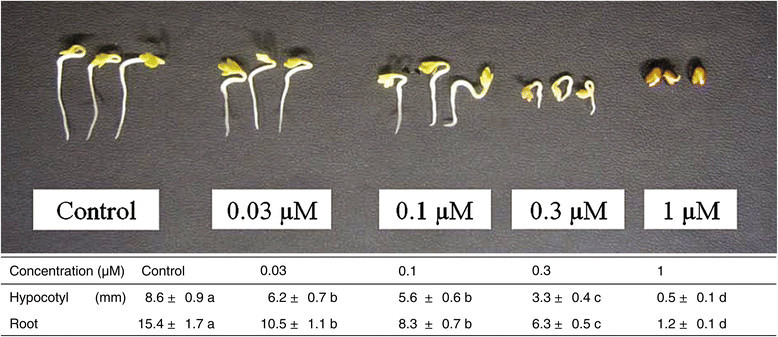
**Effects of loliolide on the root and hypocotyl growth of cress seedlings.** The different letters on the same organ indicate significant difference (*P* < 0.05) according to Duncan’s multiple comparison tests.

Loliolide inhibited root and hypocotyl growth of cress at concentration greater than 0.03 μM (Figure 5). At the concentration of 1 μM of loliolide, cress roots and hypocotyls were inhibited by 7.8 and 5.8% that of control, respectively. The concentrations of loliolide required for 50% growth inhibition (IC_50_) in the assay, as determined by a logistic regression analysis, were 0.18 and 0.15 μM for cress roots and hypocotyls, respectively. In addition, loliolide was reported to have anti-algal activity (Lu et al. [2011]), immunosuppressive activity (Okada et al. [1994]) and cytotoxic activity (Al-Mekhlafi et al. [2012]).

Many of the phytotoxic substances from the invasive plants have been reported to have multiple effects, including anti-herbivore, anti-fungal, anti-microbial and allelopathic activities (Lockwood et al. [2001]; Cappuccino and Arnason [2006]). Therefore, those phytotoxic substances may enhance competitive ability and make the plants invasive. Several other observations also suggest that some invasive plant species are allelopathic and their allelopathic substances are more toxic against other plants in the invasive areas than in the original areas of the invasive plants (Cappuccino and Arnason [2006]; Meiners et al. [2012]). In addition, phytotoxic active substances in plants can be released into the neighboring environment, either as exudates from living plant tissues or by decomposition of plant residues (Bais et al. [2006]; Bonanomi et al. [2006]; Belz [2007]).

## Conclusion

A phytotoxic substance with allelopathic activity was isolated from *C. aquatica* extract. The chemical structure of the substance was determined as loliolide. IC_50_ value for cress roots and hypocotyls was 0.18 and 0.15 μM, respectively. These results suggest that loliolide may contribute to the allelopathic effect caused by the *C. aquatica* extract.

## Authors’ original submitted files for images

Below are the links to the authors’ original submitted files for images. Authors’ original file for figure 1 Authors’ original file for figure 2 Authors’ original file for figure 3 Authors’ original file for figure 4 Authors’ original file for figure 5

## Abbreviations

IC_50_: Concentrations required for 50% growth inhibition
HRESI-MS: High-resolution electrospray ionization mass
