# Supplementary figures and images for: Isolation and identification of a phytotoxic substance from the emergent macrophyte Centrostachys aquatica
Source: Bot Stud. 2014 Aug 12;55:59. doi: 10.1186/s40529-014-0059-1 (PMC5430306; doi:10.1186/s40529-014-0059-1)

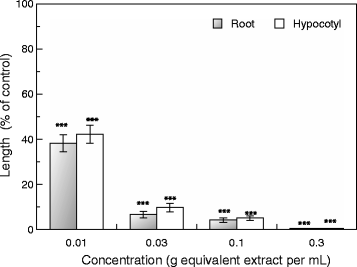

Supplement: Supplementary file 1 — Authors’ original file for figure 1 [file 40529_2014_9059_MOESM1_ESM.gif]

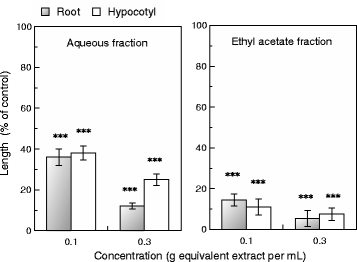

Supplement: Supplementary file 2 — Authors’ original file for figure 2 [file 40529_2014_9059_MOESM2_ESM.gif]

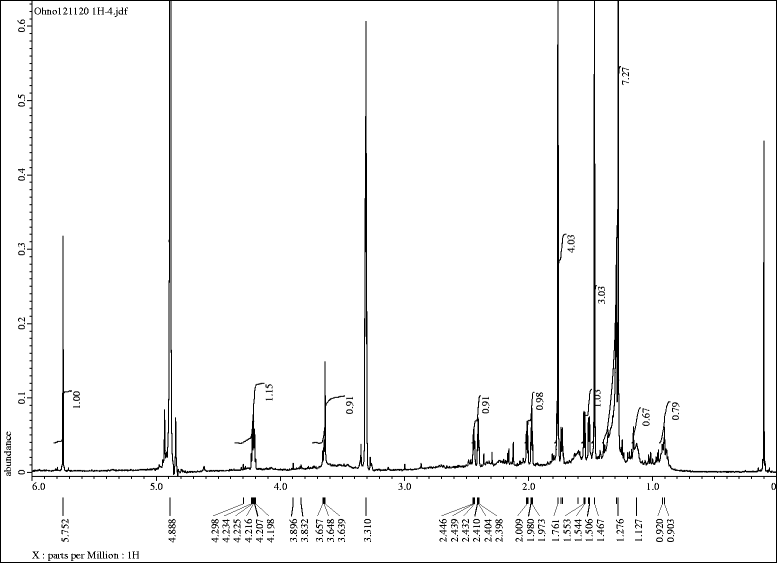

Supplement: Supplementary file 3 — Authors’ original file for figure 3 [file 40529_2014_9059_MOESM3_ESM.gif]

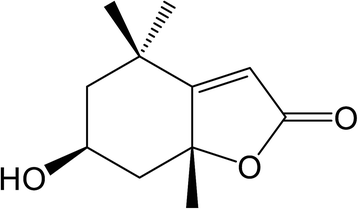

Supplement: Supplementary file 4 — Authors’ original file for figure 4 [file 40529_2014_9059_MOESM4_ESM.gif]

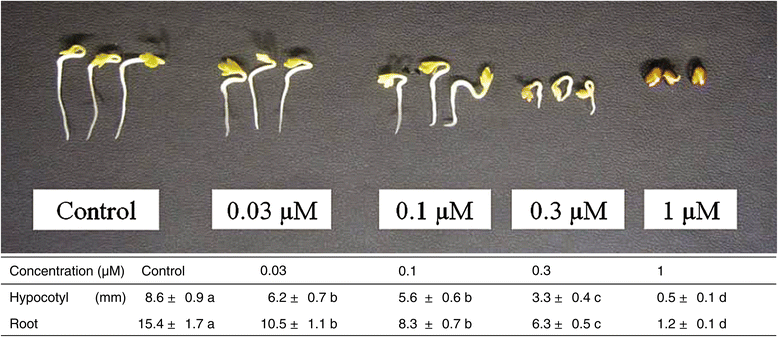

Supplement: Supplementary file 5 — Authors’ original file for figure 5 [file 40529_2014_9059_MOESM5_ESM.gif]
